# A Machine Learning Method for the Prediction of Receptor Activation in the Simulation of Synapses

**DOI:** 10.1371/journal.pone.0068888

**Published:** 2013-07-23

**Authors:** Jesus Montes, Elena Gomez, Angel Merchán-Pérez, Javier DeFelipe, Jose-Maria Peña

**Affiliations:** 1 Departamento de Arquitectura y Tecnología de Sistemas Informáticos, Facultad de Informática, Universidad Politécnica de Madrid, Madrid, Spain; 2 Laboratorio Cajal de Circuitos Corticales, Centro de Tecnología Biomédica, Universidad Politécnica de Madrid, Madrid, Spain; 3 Instituto Cajal, Consejo Superior de Investigaciones Científicas, Madrid, Spain; SUNY Downstate MC, United States of America

## Abstract

Chemical synaptic transmission involves the release of a neurotransmitter that diffuses in the extracellular space and interacts with specific receptors located on the postsynaptic membrane. Computer simulation approaches provide fundamental tools for exploring various aspects of the synaptic transmission under different conditions. In particular, Monte Carlo methods can track the stochastic movements of neurotransmitter molecules and their interactions with other discrete molecules, the receptors. However, these methods are computationally expensive, even when used with simplified models, preventing their use in large-scale and multi-scale simulations of complex neuronal systems that may involve large numbers of synaptic connections. We have developed a machine-learning based method that can accurately predict relevant aspects of the behavior of synapses, such as the percentage of open synaptic receptors as a function of time since the release of the neurotransmitter, with considerably lower computational cost compared with the conventional Monte Carlo alternative. The method is designed to learn patterns and general principles from a corpus of previously generated Monte Carlo simulations of synapses covering a wide range of structural and functional characteristics. These patterns are later used as a predictive model of the behavior of synapses under different conditions without the need for additional computationally expensive Monte Carlo simulations. This is performed in five stages: data sampling, fold creation, machine learning, validation and curve fitting. The resulting procedure is accurate, automatic, and it is general enough to predict synapse behavior under experimental conditions that are different to the ones it has been trained on. Since our method efficiently reproduces the results that can be obtained with Monte Carlo simulations at a considerably lower computational cost, it is suitable for the simulation of high numbers of synapses and it is therefore an excellent tool for multi-scale simulations.

## Introduction

Most information in the mammalian nervous system flows through chemical synapses. These are complex structures comprising a presynaptic element (usually an axon terminal) and a postsynaptic element (a dendritic spine, a dendritic shaft, an axon, or a soma) separated by a narrow gap known as the synaptic cleft. The neurotransmitter is stored in synaptic vesicles located in the presynaptic terminal. For release to take place, the membrane of one or more vesicles must fuse with a region of the presynaptic membrane, the active zone, lining the synaptic cleft. On the opposite side, the postsynaptic membrane is thickened by the presence of specific receptors and other molecules. Under the electron microscope, this area appears as an electron-dense thickening of the membrane that is known as the postsynaptic density (PSD) [1] [2]. The surface area of the active zone is proportional to the probability of synaptic vesicle release [3] [4], while the surface area of the PSD is proportional to the total number of synaptic receptors (for example, for AMPA receptors, see [5] [6] [7] [8]).

Multiple factors influence the diffusion of neurotransmitter molecules from their release to their interaction with specific receptors [9] [10] [11]. The initial concentration of the released neurotransmitter in the extracellular space depends on the volume of the synaptic cleft. The subsequent diffusion of neurotransmitter molecules outside the cleft may be influenced by the geometrical characteristics of the membranes that surround the synaptic junction. Moreover, specific transporters in the neuronal and glial membranes surrounding the synapse are involved in the rapid removal of the released neurotransmitter from the extracellular space, thereby permitting the rapid, repeated use of the synapse. However, direct observation of the various synaptic events at the molecular and ultrastructural levels *in vivo* or *in vitro* is rather difficult, if not impossible, especially in highly complex structures such as the cerebral cortex. Simulation approaches are thus useful to assess the influence of different parameters on the behavior of the synapse, such as the geometrical characteristics of the synaptic junction and its surroundings, the temperature, the presence of transporters or the number and mobility of receptors (e.g., [12], [13]).

Simulation approaches in neuroscience have considered different models, scales and techniques, according to the phenomenon being studied. Molecular dynamics are able to describe extracellular and membrane interactions or ion channel permeation [14], while some biochemical processes, such as molecular reaction-diffusion, require Monte Carlo particle-based simulators like MCell [15] [16], ChemCell [17] or Smoldyn [18] [19]. For the modeling of longitudinal ionic diffusion up to the level of neuronal circuits, some simulators such as NEURON [20] [21], GENESIS [22] or similar software (reviewed in [23]) use various approaches from simple integrate-and-fire models to highly complex Hodgking-Huxley simulations, which describe compartmental models.

Nevertheless, there are limitation issues that restrict the use of some simulation techniques. For example, current computational resources (in time and memory) prevent molecular simulation from being applied to describe full-system behavior at that scale. Some phenomena require detailed simulation at molecular level [24], which actually alters the parameters under which a larger-scale model operates. However, in other cases, many events happening on smaller scales have minimal or no effect on larger-scale processes, or, at least, they can be generalized in such a way that they can be sufficiently simplified to make their use in a larger-scale simulation feasible [25].

The field of multi-scale simulations [26] [27] [28] deals with this problem. In these approaches, the simulation, in a given scale, is generalized in the form of a simpler constructive rule that keeps the information of the key phenomena for simulation levels in a higher scale. This paper proposes the use of a machine learning method to extract the ruling patterns from a corpus of Monte Carlo simulations of synapses covering a wide range of physiological and geometrical characteristics. These patterns are later used as a predictive model of the behavior of synapses under different conditions without the need for additional Monte Carlo simulations. The use of these patterns greatly reduces the resources necessary for the simulation of this particular biological function, enabling the simulation of neuronal circuits involving thousands of different synapses, otherwise unaffordable with currently available computational resources.

## Materials and Methods

### Model synapses and Monte Carlo simulations

We have analyzed simulations based on simplified models of excitatory synapses where AMPA receptors are present and the neurotransmitter involved is glutamate. Since the number of receptors that can be found in a synapse is proportional to the area of the PSD, we have modeled synapses of different shapes and sizes to explore the influence of geometry on synaptic behavior.

**Figure 1:**
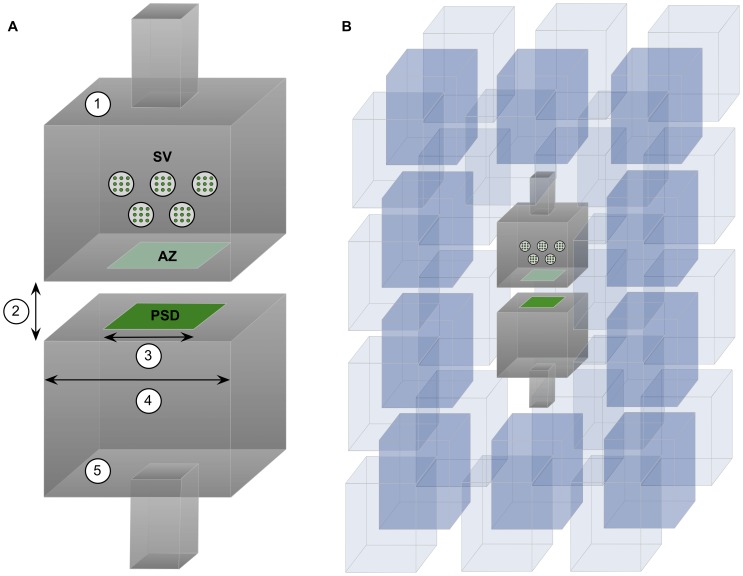
Geometrical model of chemical synapses. (A) 1. Presynaptic element containing synaptic vesicles (SV) and the active zone (AZ), at the center of which the neurotransmitter is released. 2. Synaptic cleft height. 3. Synaptic length. 4. Total apposition length. In this particular example the total apposition length is two times the synaptic length. 5. Postsynaptic element showing the postsynaptic density (PSD) where synaptic receptors are located. (B) The complete 3D geometry model composed of the pre- and postsynaptic elements of the synapse; the surrounding neuronal and glial processes; and the extracellular space. Neuronal and glial processes have been represented as polyhedral elements. The space between these elements was uniform – between 38 nm and 65 nm, depending on the size of the central synapse. The volume that represents the extracellular space was approximately 20% of the total volume.

We developed models that had a simple geometry, as far as shape is concerned, but had a variable set of parameters that specified the dimensions of the structures involved in the synaptic junction (Figure 1 and Table 1). In these simple models, the pre- and postsynaptic elements were box-shaped structures that were separated by a gap of between 15 and 20 nm (synaptic cleft height) [29]. The synapse was represented by a square with a side length (L_s_) of between 150 and 750 nm (equivalent to the cross-sectional length of the paired pre- and postsynaptic densities, see Figure 1, A). Outside the synaptic junction, the apposition of cell membranes of the pre- and postsynaptic elements extended an additional distance in all directions. The side length of the total apposition of cell membranes (E) was considered to be from 1 to 2 times the side length of the modeled synaptic junction (See Figure 1, A).

**Table 1:** Modeling and simulation parameters.

Parameter	Values
AMPA receptor density: [AMPA]	1000 to 3000 receptors per µm^2^
Glutamate transporter density: [T]	0 to 10000 molecules per µm^2^
Side length of the square representing the synapse: L_s_	150 to 750 nm
Side length of the total apposition of cell membranes: E	1 to 2 times the side length of the synapse
Synaptic cleft height: H_c_	15 to 20 nm
Glutamate molecules per vesicle	5000
Glutamate diffusion coefficient: Dg	0.4 µm^2^/ms
Time step	1 µs
Iterations	10000 (total simulation time = 10 ms)
Number of simulation runs for each model	200

Abbreviations used are also shown.

The density of receptors ([AMPA]) in the PSD was set at different levels ranging from 1000 to 3000 molecules per µm2 [8]. Glutamate transporter molecules were also modeled since the uptake of glutamate by them is essential to restore the resting level of neurotransmitter in the extracellular medium. Transporters were located on the membranes of the neuronal elements involved in the synaptic junction as well as on the membranes of other neuronal processes and glial elements located in the surrounding volume (Figure 1, B). For simplicity, we assumed that these other cells shapes were polyhedral, rather than spherical [30]. To explore the influence of the presence of glutamate transporters in cell membranes surrounding the synapse, we simulated densities of transporter molecules ([T]) ranging from the total absence of transporters to 10000 molecules/µm^2^ [31]. We adopted the glutamate transporter kinetic model and rate constants described in [32]. The volume that represents the extracellular space was approximately 20% of the total volume [33]. The distance between the extrasynaptic elements was uniform, and the cell membranes were between 38 nm and 65 nm apart ([34], [11]), depending on the size of the central synapse (Figure 1B).

**Figure 2:**
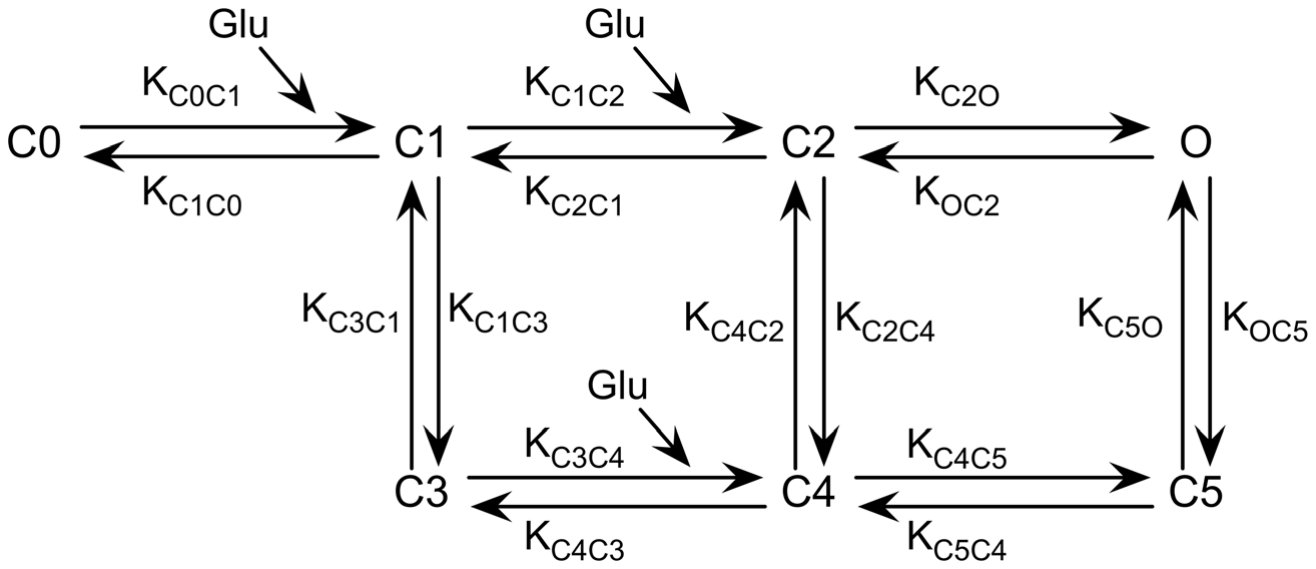
AMPA receptor kinetic model. Receptor states and rate constants were taken from [28]. Before the release of glutamate (Glu), all receptors were in the unliganded closed state (C0 state). Among the seven possible states of the receptor (C0 to C5 and O) we focused on the percentage of open receptors (O state) as a function of time since the release of glutamate.

Once the geometrical models were built, the simulations were carried out with MCell software [35], exploiting the highly optimized Monte Carlo algorithms that it uses to track the stochastic behavior of diffusing molecules. Each simulation began with the release of the content of a synaptic vesicle, which was assumed to be 5000 glutamate molecules [36]. We used a value of 0.4 µm^2^/ms as an estimation of the diffusion coefficient of glutamate (D_g_) [37] [38]. To simulate the behavior of AMPA receptors upon interaction with glutamate molecules, we adopted the kinetic model and rate constants described by Jonas et al. [39] (Figure 2). Before the release of glutamate, all receptors were in the unliganded, closed state. After release, the receptors could be found in seven possible transition states, but we focused on the percentage of open receptors as a function of time since glutamate release. Modeling and simulation parameters are summarized in Table 1.

We randomly generated a total of 1000 different models of synapses, uniformly covering all parameter ranges (Table 1). We then simulated these configurations with MCell. Each simulation consisted of 10,000 iterations with a time step of 1 µs, corresponding to a total simulation time of 10 ms after neurotransmitter release. Due to the stochastic nature of the simulations, each of the 1000 model synapses was simulated 200 times with different random seeds. The synaptic model simulations were performed using a supercomputer, the Magerit system, located at the CeSViMA [40]. Magerit is a cluster consisting of 245 eServer BladeCenter PS702 computer nodes, with a total of 3920 IBM PowerPC 3.3 GHz CPU cores and 7840 GB of RAM. The MCell developing team [35] kindly provided a version of the MCell software for the PowerPC architecture. The simulation of synaptic models involved 200,000 jobs executed on this supercomputer, requiring more than 3,500 CPU hours. Since 800 CPUs were used simultaneously, the whole set of simulations took approximately 4.5 hours.

**Figure 3:**
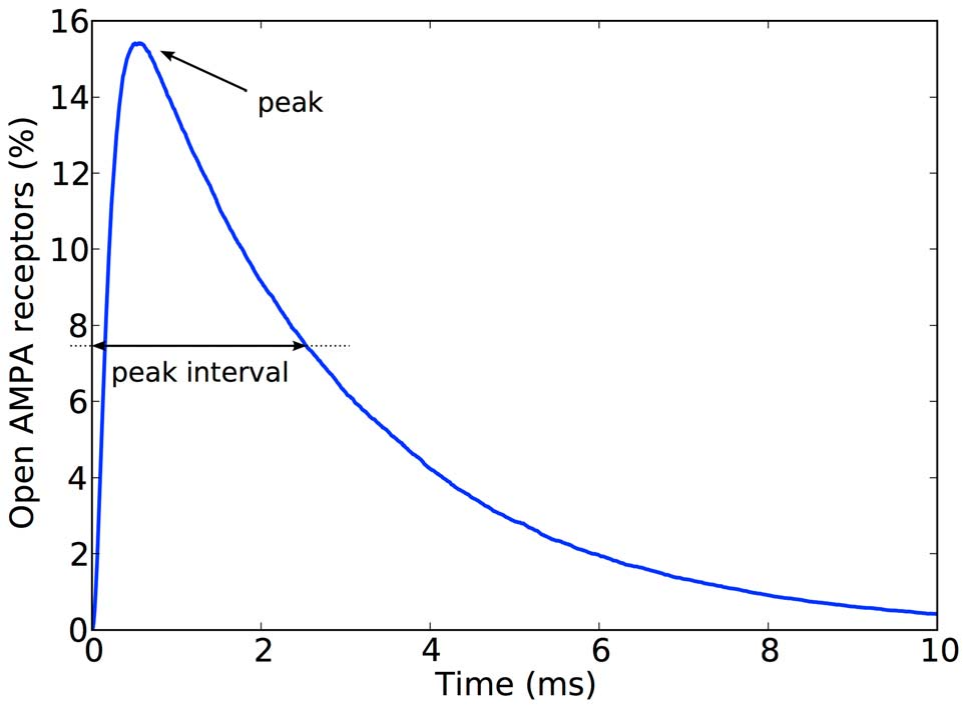
Percentage of open AMPA receptors after the release of a single vesicle of glutamate. An example of the characteristic curve obtained with Monte Carlo simulations of synapses. The peak interval contains the rapidly rising segment from the release of glutamate at t = 0 to the peak, and the descending segment to the point where the curve decreases to 50% of the peak amplitude. The tail is the rest of the simulated curve. Synapse parameter values: [AMPA] = 2000 molecules/µm^2^, [T] = 5000 molecules/µm^2^, L_s_ = 600 nm, H_c_ = 15 nm, E = 2.00.

**Figure 4:**
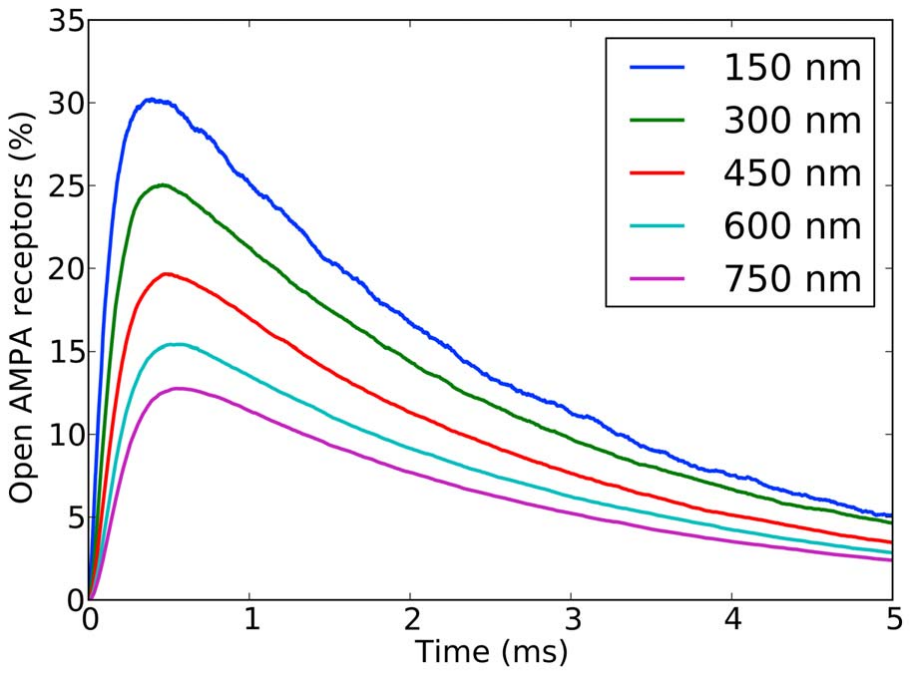
Monte Carlo simulations of synapses of different sizes. The five curves represent the percentage of open AMPA receptors after the release of glutamate in synapses of five different side lengths. These side lengths are shown in the upper right inset. All other parameters were kept constant. Each curve represents the mean of 200 Monte Carlo simulations performed with MCell. The rest of the synapse parameter values are [AMPA] = 2000 molecules/µm^2^, [T] = 5000 molecules/µm^2^, H_c_ = 15 nm and E = 2.00.

When every model synapse had been simulated 200 times, the average percentage of open AMPA receptors was plotted as a function of time since glutamate release (Figure 3). The curves obtained were consistent with previous studies, such as those presented by [32], [41], [42] and [43]. All curves show a rapid climb to a single peak followed by a slower descent, with an apparent asymptote at 0. We have referred to the initial section of the curve (containing the rapid ascent, peak and descent) as the *peak interval* and the remainder of the curve as the *tail*. The *peak interval* contains the most relevant information, i.e. the amplitude of the peak and the time it takes to reach it. These two characteristics depend on the synapse configuration. An exploratory analysis of the data showed that the smaller the active zone and PSD, the faster the peak is generally reached and a higher value is achieved. It is important to remember that these are AMPA activation percentage values, and therefore are relative to the absolute number of AMPA receptors present, which depends on the density of receptors and the size of the synapse. Figure 4 shows a comparison between the AMPA activation series obtained from synapses of different sizes.

### Receptor activation function

The experimental simulations performed with the help of MCell provided a comprehensive dataset of AMPA receptor behavior in a wide range of different synapses. For each synapse configuration, this dataset contained a unique time series (the average of 200 simulation runs) showing the evolution of the percentage of open receptors at any given time. Using this information, our main objective was to design an effective methodology for constructing a *receptor activation prediction model*. This model can be expressed as the following mathematical function:
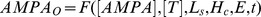
where AMPA_O_ (the average percentage of AMPA receptors that are in the open state) is a function of the concentration of AMPA receptors in the active zone [AMPA], the concentration of glutamate transporters [T], the synaptic size expressed as its side length L_s_, the cleft height H_c_, the extra space coefficient around the synapse E (the total apposition length would be E times L_s_), and the time t from glutamate release. This function would allow us to determine the average AMPA receptor activation, for any synapse, without the need to execute a new set of computationally intensive Monte Carlo simulations. It is important to note that the model is potentially capable of predicting the behavior of any given synapse provided that its physiological and geometrical parameters are known; i.e. [AMPA], [T], L_s_, H_c_, and E. Thus, this model is not merely a curve-fitting technique, but a more general model that would be able to predict the behavior of any different synapse without further adjustment to its internal parameters.

### Determining the receptor activation function

As explained before, F can be considered as a mathematical function. A first step in its definition must be to determine its mathematical form, i.e. how it can be expressed. To obtain this form, we searched for mathematical functions that could fit the curves that were obtained during the simulation process. More specifically we tested the following functions:

- Polynomial (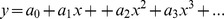): From degree 1 to 9.
- Fourier (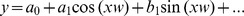): From 1 to 8 terms.
- Gauss (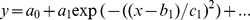): From 1 to 8 terms.
- Sum of Sine (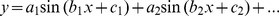): From 1 to 8 terms.
- Exponential (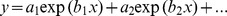): With 1 and 2 exponential terms.
- Rational (
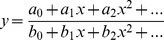
): From degree 0 to 5 in numerator and from degree 1 to 5 in denominator.

These six families of functions were selected in order to produce a comprehensive set of options from which a final model could be chosen. A total of 65 different function candidates were considered. Each function was then fitted to the average simulation curves previously obtained from each synapse configuration, using the standard Nonlinear Least Squares curve fit technique included in the MATLAB mathematical tool [44]. The results of these fits were then evaluated using the two following metrics:

- Root mean squared error (RMSE). This is one of the most commonly used measures of precision of a statistical model. RMSE is an aggregation of the individual differences (residuals) between the values predicted by an estimator and the values actually observed.
- Coefficient of determination (R^2^). This is the proportion of variability in a data set that is accounted for by the statistical model. It provides a measure of how well future outcomes are likely to be predicted by the model.

Given a reference data set Y with n values y_i_, each of which has an associated predicted value y’_i_ then the total sum of squares and the residual sum of squares are defined as:
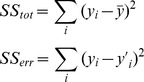



And RMSE and R^2^ metrics can be expressed as:
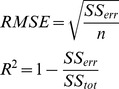



RMSE has a value equal or greater than 0, where 0 indicates a perfect fit to the reference data. R^2^ usually has a value between 0 and 1 (sometimes it can be less than 0), where 1 indicates an exact fit to the reference data and a value less than or equal to 0 indicates no fit at all. Calculating the values of RMSE and R^2^ for each curve tested provided a numerical basis to determine which function model fitted best to the synapse behavior observed.

The twelve best curve fitting results in terms of RMSE and R^2^ are shown in Table 2. RMSE and R^2^ for the 65 functions tested can be found in the Supporting Information (Table S1 in File S1). The rational model composed of a fraction of two 4-dregree polynomials and the 8-term Fourier series achieved the best results. Simpler functions of the Fourier, Gauss or exponential families (including the widely used alpha functions) yielded progressively worse metrics. We selected the best case for each function family in Table 2. The five selected candidate functions were:

**Table 2:** Best curve-fitting test results.

Curve fit	RMSE	Rank RMSE	R^2^	Rank R^2^
Rational (degree 4/4)	0.07093	1	0.99927	2
Fourier (8 terms)	0.11295	2	0.99936	1
Fourier (7 terms)	0.13507	3	0.99917	3
Fourier (6 terms)	0.16887	4	0.99876	4
Gauss (8 terms)	0.19396	5	0.99780	5
Gauss (7 terms)	0.22078	6	0.99713	6
Gauss (6 terms)	0.26144	7	0.99649	7
Fourier (5 terms)	0.30286	8	0.99604	8
Exponential (2 terms)	0.31985	9	0.98877	12
Gauss (5 terms)	0.35277	10	0.99427	9
Polynomial (degree 9)	0.41844	11	0.99241	10
Gauss (4 terms)	0.46706	12	0.99038	11

Ranked list of the 12 best curve-fitting techniques studied for the synapse MCell Monte Carlo simulation data.

- 4-by-4 degree polynomial rational function: 
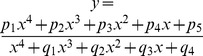
 (9 coefficients)
- 8-term Fourier series: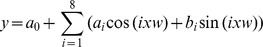 (18 coefficients)
- 8-term Gauss series: 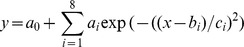 (25 coefficients)
- 2-term exponential function: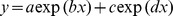 (4 coefficients)
- 9^th^ degree polynomial: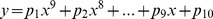 (10 coefficients)

### Estimation of function coefficients

For every one of the synapse configuration curves obtained during MCell simulations (each curve was the average of 200 runs), the coefficients of each function model were estimated by the curve fitting process provided by MATLAB. In order to directly define the AMPA_O_ function, it is necessary to establish the relationship between these values and the synapse physiological parameters ([AMPA], [T], L_s_, H_c_, and E). The main difficulty at this point was that we had 5 possible candidate functions, each with a different set of coefficients. Our preliminary objective was to obtain, for each function coefficient *p_i_*, a mathematical expression that allowed us to calculate its value in terms of [AMPA], [T], L_s_, H_c_, and E. For this task we selected a linear model of the following form:
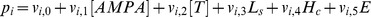



Therefore, for a function model F with a set of n+1 coefficients {p_0_, …, p_n_} and a given synapse configuration ([AMPA], [T], L_s_, H_c_ and E), the values of coefficients could be calculated as:
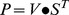
Where



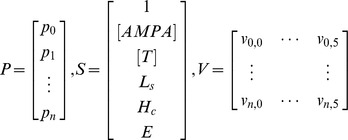



V is an n+1-by-5 matrix that contains the coefficients of the linear model. These coefficients can be calculated using a standard linear regression algorithm [45]. In order to produce accurate results from the proposed linear model, a linear relationship must exist between the function parameters and the synapse configuration coefficients ([AMPA], [T], L_s_, H_c_, and E). To determine if this was the case, we used Pearson’s linear correlation. Correlation values for every parameter of the five candidate functions can be seen in the Supporting Information (Tables S4 to S8 in File S1).

Our study revealed that none of the function candidates presented a set of coefficients where all of them have linear correlation with the synapse configuration parameters ([AMPA], [T], L_s_, H_c_, and E). This means that, regardless of the function finally selected, the AMPA_O_ function coefficients cannot be linearly obtained from the synapse configuration parameters. Therefore we needed to develop a more advanced solution to the problem of creating a general estimator of the AMPA_O_ function.

**Figure 5:**
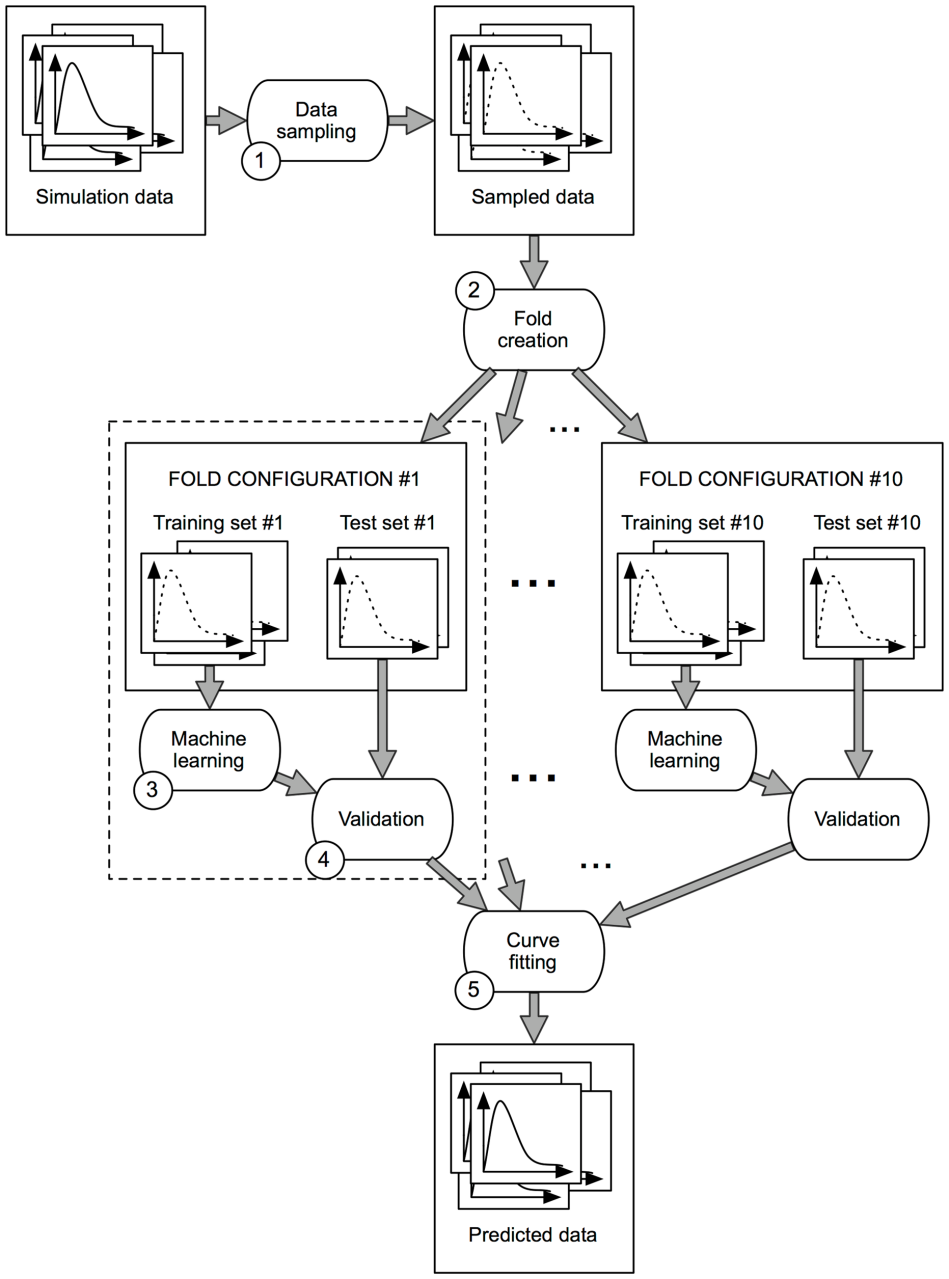
Receptor activation prediction process. The proposed method constructs a machine-learning-based prediction model of the synaptic receptor behavior in 5 distinct steps: 1. Data sampling, 2. Fold creation, 3. Machine learning, 4. Validation and 5. Curve fitting. This figure represents the main information workflow of the method.

After this preliminary study, we concluded that constructing the *receptor activation prediction model* required the use of advanced statistical analysis tools. The simulation data generated with MCell as previously described was processed and analyzed in a multi-stage process that involved tasks such as data sampling, fold creation, supervised machine learning, validation and curve fitting. A schematic representation of the entire process can be seen in Figure 5. Each of the depicted stages will now be described below in detail.

### Stage 1: Data sampling

The simulation data consisted of a set of average percentage of open AMPA receptors time series, each one corresponding to a different set of values of the synapse configuration parameters ([AMPA], [T], L_s_, H_c_, and E). Each of these time series contained information from 10 ms of simulated time, with a resolution of 1 μs. This means that each time series was composed of a set of 10,000 points. Considering that the simulation dataset contained 1000 different configurations, trying to analyze all obtained data (more than 10,000,000 points) would be extremely difficult from a computational point of view. As a reasonable alternative, the *data sampling* stage reduces the size of each AMPA time series to a set of 100 representative points. 50% of these points were selected from the curve points flanking the peak (*peak interval*), in order to maximize the amount of information obtained from the part of the series where maximum variability is observed. The rest were automatically taken from the long tail of the curve, which presents much less variability. More specifically, this sampling process is performed in the following way:

1. The *peak interval* is determined: This sub-section of the AMPA curve begins at the start of the curve, includes the peak and ends when the AMPA_O_ value decreases to 50% of the peak amplitude (see Figure 3).
2. A set of 50 points is taken from this sub-section. The sub-section duration is divided into 50 equal segments and the beginning of each of these segments is taken as part of the sub-sample to ensure all points are uniformly distributed in time.
3. A similar sampling process is performed for the rest of the curve (excluding the *peak interval*), selecting another 50 points uniformly distributed in time.

The *peak interval* is only a small part of the curve. However, the resulting curve sample will contain a lot of information about this sub-section. The reason for this is that this sub-section contains the most relevant information about the behavior of AMPA receptors, since it is the one that shows most of their activity. This more sophisticated way of performing the curve sampling (*oversampling*) ensures that information is preserved throughout the sampling process. The resulting small set of 100 chosen points contains the most relevant information regarding the open AMPA receptors' behavior and its size is much more convenient for further statistical analysis.

### Stage 2: Fold creation

Once the simulation dataset had been sampled, a machine learning process was applied, aimed at training a supervised learning model capable of predicting the average percentage of open AMPA receptors for a given synapse configuration. In a general sense, supervised learning is the process of mathematically extracting a pattern or function that explains a series of target values (e.g. a curve) present in a set of supervised training examples (e.g. a set of observed values related to the target curve). Each example is normally a pair consisting of a vector of input values and a desired output value (e.g. the observed values and the related value of the target curve). The resulting mathematical model can be a *classifier* (if the target values are discrete) or a *regression function* (if the target values are continuous). If the learning process is successful, the resulting model becomes a *predictor* of the target values. The *learning algorithm* used defines the way that this process is performed. In the method presented here, the different AMPA behavior series were used as training examples. The synapse configuration parameters and the time instant were the input vector ([AMPA], [T], L_s_, H_c_, E, t) and the open AMPA percentage was the desired output value.

Directly training a supervised learning model using all simulation data available can, however, cause an undesired effect: *overfitting*. When a machine learning prediction model is trained using a single dataset, it is possible that random error noise present in that particular set will be described by the resulting model, instead of the relevant underlying relationships between the data. When a model is *overfitted* to its training data it generally has poor predictive capabilities, as it is only able to describe the particular examples already present in the training dataset. One of the most common techniques employed to avoid this undesired phenomenon when training supervised learning models is the use of a stratified 10-fold cross-validation [46]. This technique consists of dividing the input dataset into 10 subgroups of equal size (called folds) and using them to generate 10 separate fold configurations. In each configuration, one of the folds (different in each one) is used as a *test set* and the remaining 9 as a joint *training set*. For each fold configuration the machine learning model is trained using the *training set*. The same model is then validated (checked to determine whether it can correctly predict the output value) using the *test set*.

The AMPA activation prediction method performs a typical 10-fold cross-validation process such as the one described above. The 1000 synapse configurations available were separated into 10 groups of equal size and the corresponding fold configurations were generated, including a different *training set* and *test set* for each fold. These configurations were then used in the next stages of the process.

### Stage 3: Machine learning

To generate the AMPA receptor behavior prediction model during the machine learning stage, several regression algorithms were tested:

- Linear regression. The aim of a regression analysis [45] is to determine the statistical relation that exists between a dependent variable and one or more independent variables. A functional relation between the variables must be postulated, and a linear curve is fitted to the data.
- The K-Nearest Neighbors algorithm (KNN) [47] is a classifier/regression algorithm based on agreement. When used for regression, an object is assigned to a weighted average of its k nearest neighbors in the training set.
- A Multi-Layer Perceptron (MLP) [48] is an artificial neural network model that selects the corresponding output for the specific input data. The MLP extends the standard linear perceptron using several layers of neurons. It can be used both as a classifier and regression technique, depending on the input variables.
- M5 [49] is an algorithm that generates a decision model in the form of a tree. This algorithm builds trees whose leaves are associated with multivariate linear models and the nodes of the tree are chosen over the attribute that maximizes the expected error reduction as a function of the standard deviation of output parameter. More specifically, a M5P variant [50] was considered in the present study. These model trees can be easily converted into regression rules.
- Multivariate adaptive regression splines (MARS) are a form of regression analysis introduced by Jerome Friedman in 1991 [51]. They are non-parametric regression techniques and can be seen as an extension of linear models that automatically model non-linearities and interactions between variables.
- Projection Pursuit Regression (PPR) [52] is a method for non-parametric multiple regression. It is more general than standard stepwise regression procedures, does not require the definition of a metric in the predictor space, and lends itself to graphical interpretation.

These machine learning algorithms were selected in order to perform a study that was as comprehensive as possible. The six techniques presented are well known, widely used and scientifically relevant. All of these where tested using the available data from the 1000 synapse configurations in order to determine the most suitable machine learning technique for the problem at hand. The performance of classification/regression algorithms always depends greatly on the characteristics of the data to be analyzed, and there is no single algorithm that produces optimal results for any given problem. This phenomenon can be explained by the *no free lunch* theorem, which states “any two learning algorithms are equivalent when their performance is averaged across all possible problems” [53]. Using the selected machine learning algorithms, a regression model was trained from the training set of each fold configuration. The accuracy and correctness of these models were then evaluated in the next stage.

### Stage 4: Validation

Once the algorithms had been trained, their correctness was validated using the *test set* of each fold configuration. For this purpose we used the two validation metrics previously described (RMSE and R^2^). The input vector of each point in the *test set* is introduced in the prediction model. The result is then compared to the expected value. Once all points are predicted, the RMSE and R^2^ metrics are calculated. Detailed results of these tests can be found in the Supporting Information (Tables S2 and S3 in File S1). The M5P algorithm produced the best results according to both performance metrics (See Table 3 and Results section).

**Table 3:** Validation stage results.

Regression technique	RMSE	R^2^
M5P	0.6357	0.9808
KNN	0.8875	0.9774
MLP	18.812	0.8722
PPR	24.231	0.8255
Linear Regression	31.067	0.7235
MARS	31.077	0.7234

The average value of each validation metric for each regression technique is shown, sorted from best to worst. The metrics are calculated for each case by comparing the initial curve sample of 100 points with the corresponding 100 predicted points obtained using each regression algorithm. M5P obtained the best results with both RMSE and R^2^ metrics.

### Stage 5: Curve fitting

At the end of the validation stage, the process produced a series of points and two precision metrics. The points are the predicted values for the percentage of open AMPA receptors at each instant of time selected in data sampling stage 2. The precision metrics indicate how accurate this prediction is.

Assuming that these prediction metrics show acceptable values, the fifth stage attempts to infer a mathematical function capable of determining the percentage of open AMPA receptors for any given time instant. As previously described, several function models where tested in order to find the most suitable match (polynomial, Fourier, Gauss, sum of sine, exponential, and rational). At this point we selected the two best previously studied models: *i)* a rational polynomial model (the best function model according to RMSE) and *ii)* a Fourier series (the best function model according to R^2^) of the following form:
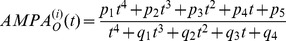


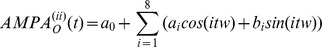



The first case involves a fraction of two 4-degree polynomials, containing a total of nine coefficients. The second case is an 8-term Fourier series with 18 coefficients. Using the predicted points obtained from the validation stage, it is possible to calculate the values of these coefficients by means of an automatic curve-fitting process. This curve fitting was performed using the MATLAB curve-fitting tool. The precision of this process was again measured using the two metrics indicated in validation stage 4.

Ultimately, only one of these two AMPA_O_ models (either the rational function or the Fourier series) is necessary. Therefore, in order to determine the best performing one, it was necessary to evaluate the final results of this curve-fitting stage. These results are presented and discussed in the following section.

The entire *AMPA activation prediction* process was implemented using the C++, Python and Java programming languages and the MCell Description Language (MDL).

The five stages of the *receptor activation prediction* process were developed as a set of configurable programs written in Python, Java and C++. For the supervised machine learning tools and algorithms, Weka data mining open source software was used [46]. Other mathematical and programing tools used include R [54], MATLAB [44] and the NumPy and SciPy libraries.

The five-stage receptor activation prediction process was executed on a regular desktop computer with a 4-core Intel i5 2.4GHz CPU and 4GB of RAM. The process was carried out in separate stages, with a total aggregated computation time of less than 5 hours. Software can be downloaded from http://cajalbbp.cesvima.upm.es/ampaprediction and ModelDB (http://senselab.med.yale.edu/ModelDB/showmodel.asp?model=150207).

## Results

### Machine learning validation results

During stages 2 and 3 of the *AMPA activation prediction* process, the simulation dataset was divided into 10 fold configurations that were used during the machine learning process. Stage 4 was concerned with the statistical evaluation of the results of these processes. For each fold configuration, the RMSE and R^2^ metrics were calculated after using the M5P algorithm, producing the results shown in Table 3.

As can be seen, all metrics provided excellent results. The M5P algorithm seems to be a very suitable technique for the task at hand, capable of very accurately predicting the values of the average percentage of activated AMPA receptors. All fold configurations showed very close-fitting values both for RMSE and R^2^, and the aggregated results (containing the predicted values for all synapse configurations samples) were equally good. The R^2^ metric was especially interesting since it is the more sophisticated one, and is especially appropriate for prediction assessment. In this case the average value was above 0.98, indicating an almost exact fit to the test data (a value of 1 would indicate an exact prediction). This is especially relevant since, as described in the Materials and Methods section, the 10-fold cross-validation process ensures that no information from any synapse configuration is used to train the part of the machine learning model that predicts it. This seems to indicate that the M5P algorithm has been able to avoid overfitting and has been capable of inferring the underlying relations between the synapse configuration characteristics, the time elapsed since glutamate vesicle liberation and the AMPA receptors activation.

It is important to remember that these results are related to the validation stage of the *receptor activation prediction* process, and therefore are obtained from the sampled simulation values only (100 per synapse configuration).

### Final prediction results

**Figure 6:**
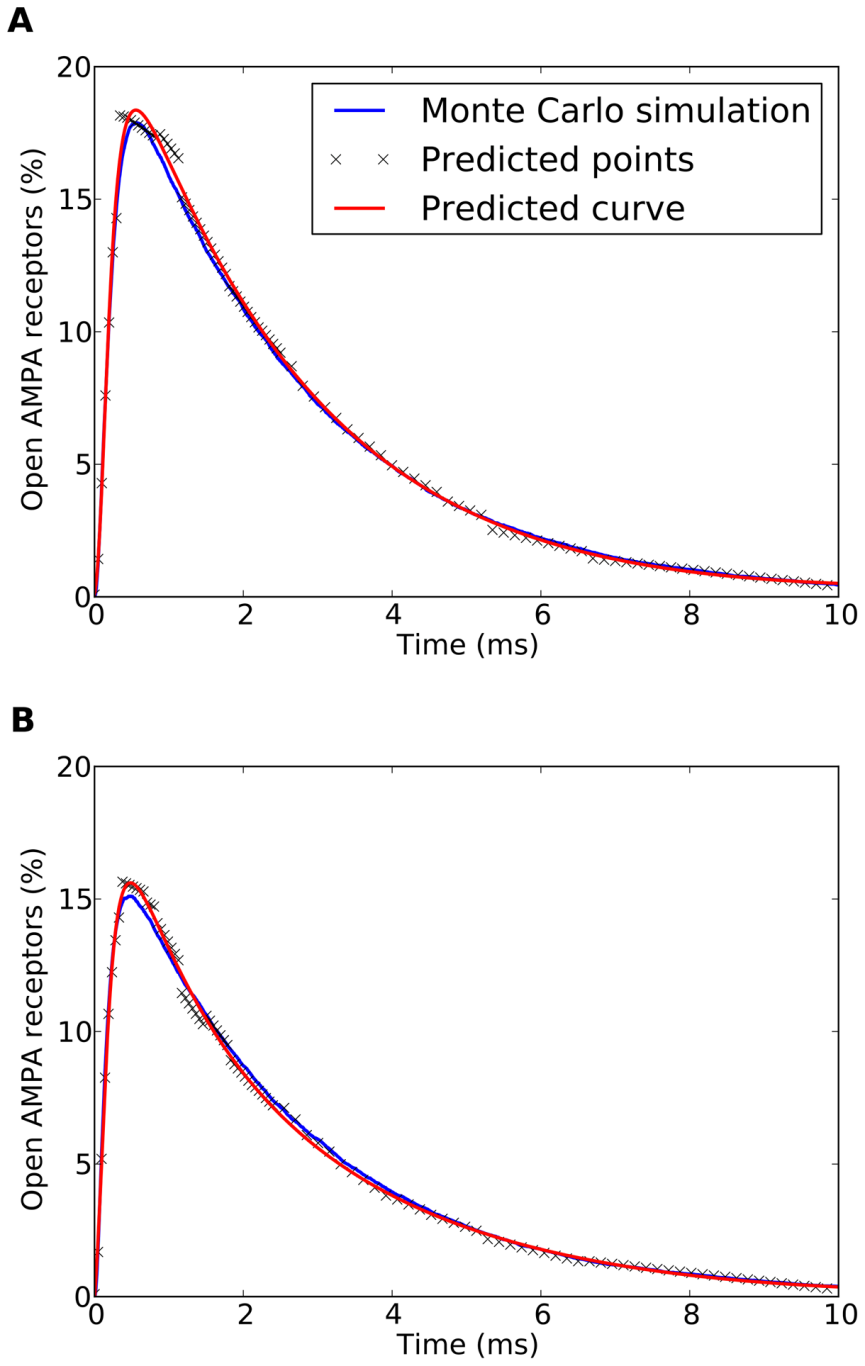
Predicted receptor activation curves. Two examples of predicted curves (fitted using the rational model) compared with the experimental curves obtained by Monte Carlo simulations. The Monte Carlo simulation curves (blue traces) are the mean of 200 simulations performed with MCell. The 100 predicted points per curve (small crosses) were obtained in the machine learning validation stage. These points were later used to fit the predicted curve (red traces). (A): [AMPA] = 1614 molecules/µm^2^, [T] = 508 molecules/µm^2^, L_s_ = 534 nm, H_c_ = 18 nm, E = .65. The error metrics for the final predicted curve were: RMSE = 0.2058, R^2^ = 0.9984. (B): [AMPA] = 2878 molecules/µm^2^, [T] = 9155 molecules/µm^2^, L_s_ = 456 nm, H_c_ = 16 nm, E = 1.36. The error metrics for the final predicted curve were: RMSE = 0.1857, R^2^ = 0.9981.

After the machine learning validation had taken place, the final curve fitting stage was performed. This made use of the 100 points predicted for each synapse configuration to infer the entire series of open AMPA receptors (AMPA_O_). As explained previously, this series was fitted to the rational and Fourier models presented in the Materials and Methods section. The results of this curve fitting process were evaluated using the RMSE and R^2^ metrics. In this case, all the original values of each AMPA curve were compared to the corresponding mathematical function obtained after the curve fitting process. This gave a final measurement of the AMPA prediction capabilities of the method presented, since the final estimated curve was compared to the original experimental data. Figure 6 shows two examples of this final predicted curve, compared with the Monte Carlo simulated experimental series obtained with MCell. The figure also shows the 100 predicted points per curve obtained in the machine learning validation stage, that were used afterwards to fit the final receptor behavior prediction curve.

The same fitting process was performed for both curve models (rational and Fourier) and all 1000 synapse configurations, and the precision metrics were calculated. Table 4 shows the final mean and standard deviation values observed for those metrics. Detailed results can be seen in the Supporting Information, Table S9 in File S1.

**Table 4:** Final prediction results.

	Mean (Rational)	Stdev (Rational)	Mean (Fourier)	Stdev (Fourier)
**RMSE**	0.3122	0.4537	0.3252	0.4387
**R^2^**	0.9914	0.04789	0.9929	0.0340

Final AMPA receptor activation prediction results obtained using either the 4-by-4 degree polynomial rational function or the 8-term Fourier series as curve-fitting models.

**Figure 7:**
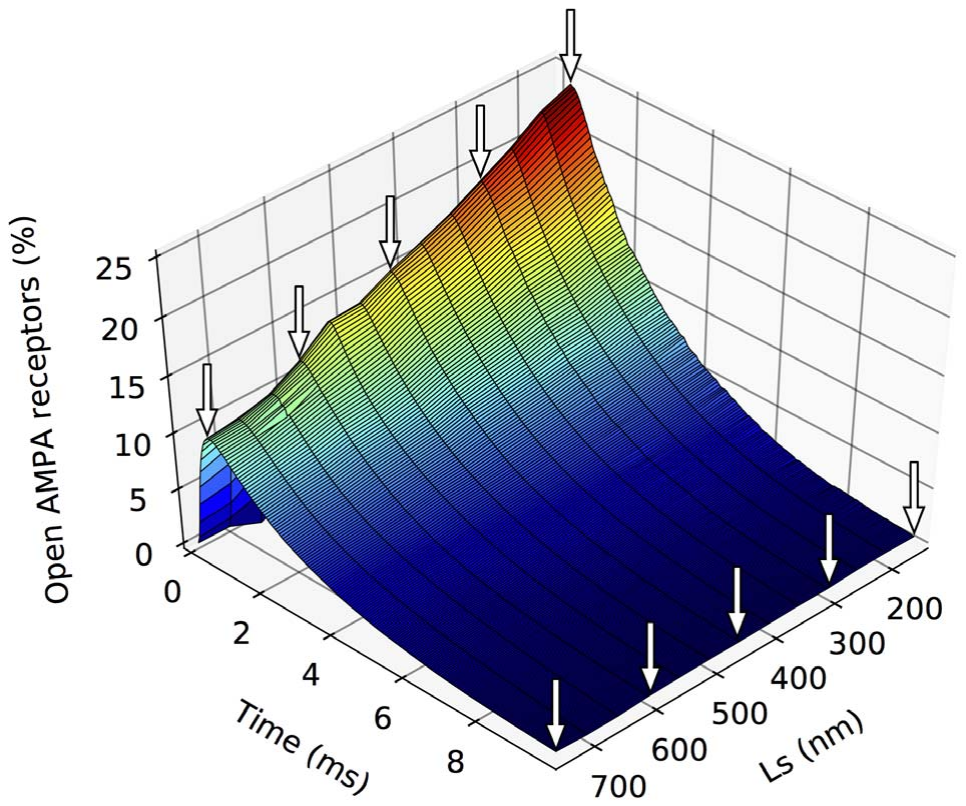
Comparison between predicted and Monte Carlo simulated curves of the percentage of open AMPA receptors. The figure shows 13 curves where all synapse parameters were kept constant except the side length of the synapse (Ls). Five curves (marked with arrows) were generated by Monte Carlo simulations to serve as references (Ls = 150 nm, 300 nm, 450 nm, 600 nm and 750 nm). The rest of the synapse parameter values were [AMPA] = 2000 molecules/µm^2^, [T] = 2500 molecules/µm^2^, Hc = 20 nm and E = 1.75.

Results show that, using the rational function, the final *AMPA activation prediction* model provides a very accurate estimation of the average percentage of active AMPA receptors curves. Both precision metrics (RMSE and R^2^) present excellent results, validating the quality of the prediction model and demonstrating its desired characteristics. The resulting model is capable of predicting the average AMPA receptor activation curve for any synapse configuration whose parameters are within the range of the synapses originally simulated using the Monte Carlo method. Thus, curves representing continuous changes in synapse parameters can be generated (Figure 7). The Fourier series seems to produce equally accurate curves, but at the cost of having a much more complex model (18 coefficients against 9 in the rational model). For this reason we do not recommend the use of the Fourier model for this stage, although its numerical results are equally good.

Not all synaptic parameters have the same impact on the average percentage of activated AMPA receptors. To determine which of these parameters are the most influential in our simulations, we calculated the Pearson’s linear correlation coefficient of each synaptic parameter with the average peak amplitude of the percentage of activated AMPA receptors. The most influential parameter, revealed by the highest (inverse) correlation, was Ls, with a coefficient of −0.795. E and Hc yielded much lower coefficients of 0.330 and −0.305, respectively, followed by [T] and [AMPA], with −0.260 and −0.008, respectively (all these values are shown in Table S10 in File S1). We also evaluated the possibility that for certain values of the variables, our method would give better results than for other values. To do this, we plotted the distribution of RMSE and R^2^ error values against the values of the five variables used in the model synapses (see Table S9 in File S1), and we found no evidence of clustering of good (or bad) error values around any variable values (see Figures S1 and S2 in File S1).

### Extendibility of the prediction model

Results show that the method presented in this paper can generate a very accurate AMPA receptor activation prediction model based on a series of synaptic Monte Carlo simulations, using five different variables ([AMPA], [T], Ls, Hc and E). This is, of course, a simplified model of the synapse, and other variable parameters could be taken into account, depending on the specific interests of the researcher using our method. The techniques described in this paper are designed to be easily adapted, so new synaptic parameters can be readily incorporated into the model. Moreover, since the method includes its own evaluation mechanisms, it provides a measurement of the accuracy of the prediction model for the synaptic parameters selected.

To illustrate this extendibility, we performed an additional series of experiments, increasing the number of variables to 6. We kept the five original variables, and added a new one: the diffusion coefficient of glutamate, Dg, which had previously been considered constant. For this new series, we generated 2000 new synaptic configurations, randomly varying the five original synaptic variables within the same ranges used in the previous experiments (see Table 1 for details). The new variable Dg, was randomly sampled between 0.25 and 0.75 µm^2^/ms [55] [56] [57]. The sample size for Monte Carlo simulations was doubled (from 1000 configurations to 2000) to exhaustively cover all new data variability due to the introduction of the additional variable. Using this new experimental dataset, we performed the five stages of our method, as in the case of the original simulation dataset. Error metrics are summarized in table 5. Detailed results can be found in the Supporting Information, Table S11 in File S1.

**Table 5:** Final prediction results with an extended scenario.

	Mean (Rational)	Stdev (Rational)	Mean (Fourier)	Stdev (Fourier)
**RMSE**	0.7221	3.4980	0.6167	1.8710
**R^2^**	0.9643	0.1214	0.9728	0.0930

Summary of the AMPA receptor activation prediction results obtained using either the 4-by-4 degree polynomial rational function or the 8-term Fourier series as curve-fitting models. In these experiments, an extended simulation dataset was used, including six different synaptic variables. The new variable included was the diffusion coefficient of glutamate.

The model accuracy was still very high, although lower than with the original experimental series. This was most probably due to the increased complexity arising from the addition of a new variable. In this case, the Fourier fit produced better results than the rational fit with both accuracy metrics. Therefore, in this particular case we would recommend the use of this technique in stage 5 of our method. This example illustrates how the incorporated accuracy metrics can help our method to achieve the best possible results.

## Discussion

In this paper we have demonstrated the difficulties of constructing an accurate automated predictor of the behavior of Monte Carlo simulations of synaptic receptors in synapses with a wide range of different structural and physiological characteristics. Nevertheless, we have developed an advanced synapse behavior modeling process that is capable of achieving this goal.

The entire process described in the present article is performed automatically. The extensive range of synaptic structural and physiological configurations to be explored in order to generate a comprehensive synapse receptor behavior model requires a method where the neuroscientist is only concerned with the relevant aspects of the experimental configuration and results, relying on the computer to perform all the sophisticated data handling and mathematical analysis. A computer, using standard statistical software tools, can perform all five stages by itself and no human supervision is required once the initial simulation data have been gathered.

The *prediction* mechanism used in the present study is generic, which means that it does not work by simply “memorizing” its training data and afterwards recalling the corresponding information when asked about a previously simulated synapse configuration. On the contrary, it is capable of extracting knowledge and learning highly complex patterns that describe how synapse receptors behave under different conditions. It applies this knowledge afterwards, when required to predict a new, not previously simulated synapse configuration. The nature of the 10-fold cross-validation process guarantees that no data from any specific synapse configuration is used when training a model to predict it (since it cannot be in the *training set* and *test set* at the same time). This avoids overfitting and makes the generation of more general prediction models possible.

Furthermore, once the prediction model is created, no further experimental Monte Carlo simulations are required. Since the prediction model is able to extrapolate results other than those used for learning, this model can be used in place of experimental simulations. Of course, the generic nature of the receptor prediction model will strongly depend on the quality of the initial experimental data used to train it. This data has to be sufficiently rich in order for the machine learning process to be able to learn and extract useful synapse behavior patterns. In this paper we have explored a wide range of different structural and physiological synapse characteristics in order to create a comprehensive training set. With this requirement fulfilled, the prediction function effectively replaces the average percentage of open receptors observed by a series of experimental simulations, which would be much more computationally expensive. Therefore, from an experimental perspective, the receptor behavior prediction model represents an excellent tool, since it drastically reduces the computational cost of determining this average receptor behavior.

**Figure 8:**
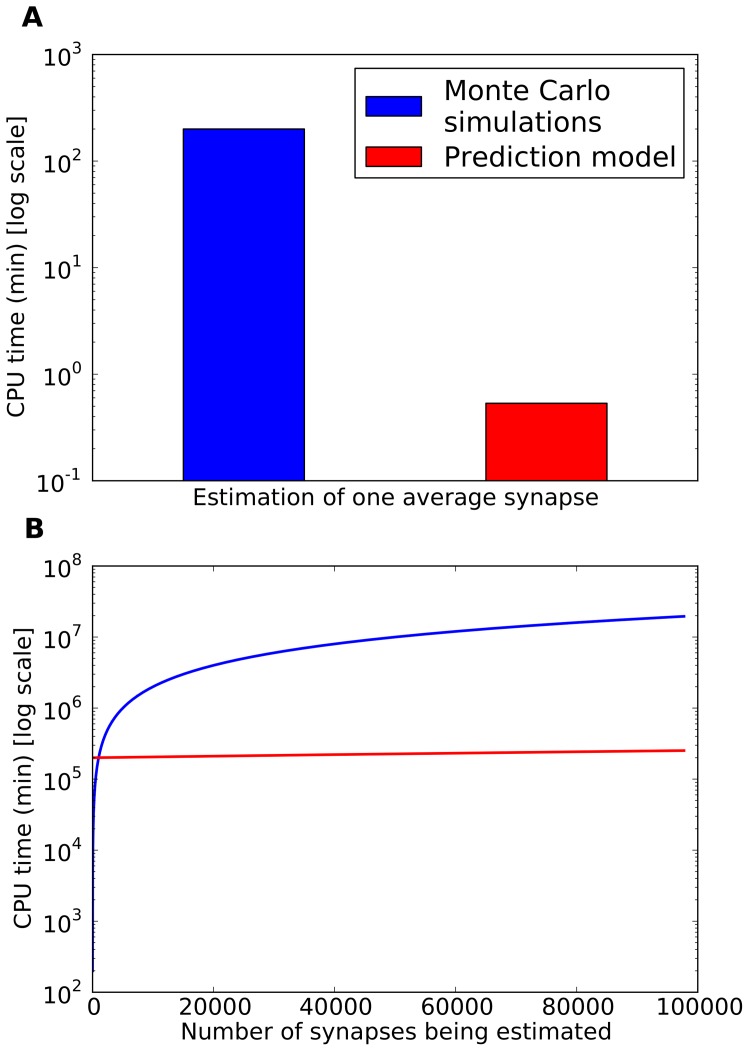
Comparison of computational costs of synapse Monte Carlo simulations vs. the proposed AMPA prediction model. CPU time required for obtaining statistically representative average AMPA receptor activation information (mean of 200 stochastic simulations) using both methods on a regular desktop computer. (A): Comparison between the CPU cost of estimating average behavior of a single synapse. (B): Linear extrapolation of the CPU time required to model an increasingly large number of synapses. This includes the initial set of Monte Carlo simulations (1000 synapse configurations) required to train the machine learning model. The prediction model CPU time curve growth is barely perceptible due to the great slope difference with the Monte Carlo simulations curve (the Monte Carlo simulations CPU time grows approximately 400 times faster). In both cases, CPU time is presented in logarithmic scale.

To better understand the magnitude of this improvement, it is important to consider the time spent and computational resources used during the development of this technique. To create the initial training set a total of 200,000 synapse simulations were executed using MCell (1,000 synapse configurations, 200 executions per configuration). These simulations were carried out on the Magerit supercomputer, using 800 CPU cores and amounting to a total of over 3,500 aggregate CPU hours, or approximately 1 min/simulation on average (since 800 CPU cores were used in parallel, the whole set of simulations took approximately 4.5 hours). In contrast, once the receptor activation prediction model presented here has been trained, it only requires approximately 8 CPU seconds to predict the average behavior of a specific synapse on a regular 4-core desktop computer (Intel Core i5 2.4Ghz), that is, a total of 32 seconds of CPU time (8 seconds ×4 cores). Our receptor activation prediction technique would require only 8.9 hours (32,000 seconds) of aggregated CPU time to generate 1,000 synapse configurations (the same number that were generated using MCell). Therefore, the use of this technique maintains the accuracy of Monte Carlo simulations (for the range of parameters that we have considered) reducing the computational cost from 3,500 to 8.9 CPU hours, thus reducing the CPU total time by a factor of approximately 1/400^th^. This is an important achievement since there are trillions of synapses in the brain. For example, only one mm^3^ of human cerebral cortex contains around 10^9^ synapses [58]. Thus, the simulation of even a small portion of the brain would require a cumbersome computational effort, especially if different conditions, such as developing vs. adult, or normal vs. pathological nervous tissue need to be modeled and compared. With our method, large numbers of different synapses can be simulated using a regular computer. Indeed, as mentioned above, a regular 4-core desktop computer can generate the average behavior of one synapse in 8 seconds, that is, 450 different synapses per hour. If our technique were implemented in a supercomputer such as Magerit, using 800 processing cores, the number of simulated synapses would increase at least by two orders of magnitude, to 90,000 per hour or more. Thus the simulation of thousands of millions of synapses present in the brain would be feasible by incrementing computation time and power. For example, future availability of exascale computers (with hundreds of thousands or even millions of processing cores) will represent an important advance in the simulation of synapses in the whole brain. The computational benefits of our methodology are summarized in Figure 8.

It is important to acknowledge that the receptor activation prediction model cannot be trained without previously generating the Monte Carlo simulation dataset. The great advantage of the present method is that it is only necessary to perform this large set of simulations once. After carrying out the initial training that we have presented in this paper, the prediction model is capable of estimating the behavior of synapses that have not been previously simulated, so new Monte Carlo simulations are not required. In this way, an arbitrarily large population of synapses with different parameters can be modeled, provided that these parameters are within the range used in the initial Monte Carlo simulations. Alternatively, the influence of the variation of a given parameter on the behavior of individual synapses can also be modeled. For example, it is possible to predict the AMPA receptor activation curve of a population of synapses whose sizes and AMPA receptor densities are within the ranges used in this study. The same data can be used to explore the influence of variations of size and/or receptor density on individual synapses during development, as well as in plasticity or pathological circumstances. In the present work, the data generated with Monte Carlo simulations yielded the evolution of AMPA receptor states over time in a set of simulated synapses of different characteristics. We considered the peak amplitude of open AMPA receptors as a relevant parameter and our method has consequently focused on this parameter, trying to predict its value for different synaptic configurations. In principle, nothing precludes the use of this method for the prediction of other aspects of synaptic function. For example, the area under the curve of open AMPA receptors; the concentration of glutamate within the synaptic cleft at a given time point; and the evolution of any other AMPA receptor state could also be predicted from the same set of Monte Carlo simulations using the same method. However, it is not possible to know a priori how accurate the predictions will be, or how many initial Monte Carlo simulations will be necessary. Although this is certainly a disadvantage, our method does incorporate its own accuracy metrics to allow the user to evaluate new prediction scenarios.

In addition to the benefits already outlined, the low computational cost of this method and its accuracy makes it especially useful in the field of multi-scale simulations. In recent years, biology has adopted these kinds of simulations to deal with problems that cannot be described, at least not easily, with a single-scale modeling technique [26] [59] [60] [61]. Multi-scale simulations (in any of the fields they have been applied) are divided into two main categories (i) *Sequential* (also known as serial, implicit or message passing) and (ii) *Concurrent* (parallel or explicit) [25] [62]. *Sequential multi-scale simulations* define a hierarchy of modeling techniques in which the small-scale models working on highly-detailed elements provide information to construct large-scale models that deal with coarse-grain representations. Parallel multi-scale simulations bring together methods that operate at different scales in a combined approach. The simulations of these different scales are run simultaneously, exchanging information between them.

Our method can be considered as a sequential multi-scale simulation technique since a set of individual synapses are first simulated with MCell at the microsecond/nanometer scale. These simulations are then used to extract general principles governing the behavior of synapses. Using this information, relevant characteristics of synapses can finally be predicted for new synapses without the need for new Monte Carlo simulations and at a much lower computational cost.

## Supporting Information

File S1 Contains: **Figure S1. Final prediction results compared to the synaptic parameters (rational model).** Comparison between prediction errors obtained using the 4-by-4 rational curve fitting model and the different synaptic parameters. (A): Comparison to RMSE. (B): Comparison to R2. **Figure S2. Final prediction results compared to the synaptic parameters (Fourier model).** Comparison between prediction errors obtained using the Fourier curve fitting model and the different synaptic parameters. (A): Comparison to RMSE. (B): Comparison to R2. **Table S1. Curve fitting test results.** Average curve fitting test results for all possible curve fitting alternatives tested against the Monte Carlo simulation data. **Table S2. Machine learning techniques evaluation results: RMSE.** Comparison of validation results (RMSE) obtained during the 10-fold cross-validation process for all machine learning techniques tested. **Table S3. Machine learning techniques evaluation result: R2.** Comparison of validation results (R2) obtained during the 10-fold cross-validation process for all machine learning techniques tested. **Table S4. Linear correlation between synapse parameters and function coefficients: 4-by-4 degree polynomial rational function.** Observed linear correlation between synapse parameters and coefficients of the 4-by-4 degree polynomial rational function for all Monte Carlo synapse simulations. **Table S5. Linear correlation between synapse parameters and function coefficients: 8-term Fourier series.** Observed linear correlation between synapse parameters and coefficients of the 8-term Fourier series for all Monte Carlo synapse simulations. **Table S6 Linear correlation between synapse parameters and function coefficients: 8-term Gauss series.** Observed linear correlation between synapse parameters and coefficients of the 8-term Gauss series for all Monte Carlo synapse simulations. **Table S7. Linear correlation between synapse parameters and function coefficients: 2-term exponential function.** Observed linear correlation between synapse parameters and coefficients of the 2-term exponential function for all Monte Carlo synapse simulations. **Table S8. Linear correlation between synapse parameters and function coefficients: 9-degree polynomial.** Observed linear correlation between synapse parameters and coefficients of the 9-degree polynomial function for all Monte Carlo synapse simulations. **Table S9. Final prediction results.** Comparison between Monte Carlo simulations and prediction results obtained for all 1000 synapse simulations, using the 4-by-4 rational and Fourier curve fitting models. (A): Detailed results. (B): Average and stdev values. **Table S10. Correlation between synaptic parameters and AMPA activated receptors curve peak.** Pearson's linear correlation coefficient between the AMPA activated receptors curve peak and the values of the different synaptic parameters. **Table S11. Final prediction results of the extended experiment.** Comparison between Monte Carlo simulations and prediction results obtained for all 2000 synapse simulations, using the 4-by-4 rational and Fourier curve fitting models. (A): Detailed results. (B): Average and stdev values.
(XLSX)
